# Personal

**Published:** 1909-04

**Authors:** 


### PERSONAL.

---

Dr. Frederick W. Koehler of 582 Broadway, Buffalo, has been appointed an inspector of food and drugs in the Health Department and has been assigned to inspect eight or nine small slaughterhouses in the city which have not been heretofore inspected.

Dr. Koehler was certified by the Civil Service Commission and he was the only one on the list, with a percentage of 90.88. eight other doctors taking the examination having failed to pass.

---

Dr. Gustav A. Hitzel, of Buffalo, was re-elected president of the German-American Alliance at the recent annual meeting of the delegates, representing 7,500 members. Under Dr. Hitzel's administration the organisation has greatly increased in membership and enthusiasm, and has successfully undertaken important work.

---

Dr. Arthur Le Roy Pulver, late house surgeon at the Buffalo General Hospital, and Mrs. Pulver, left Buffalo, recently to reside at Wellsville, N. Y.

---

Dr. George F. Cott, of Buffalo, read a paper at a recent meeting of the Medical Society of the County of Ontario held at Canandaigua. Dr. Cott's subject was "Acute Endolabyrinthitis with Autointoxication."

